
JOURNAL INFORMATION
==============================
NLM Title Abbreviation: ScientificWorldJournal
Journal ID: ScientificWorldJournal
NLM Journal ID: 101131163
Journal ID: TSWJ

The Scientific World Journal

ISSN: 2356-6140
EISSN: 1537-744X
Publisher: Wiley

ARTICLE INFORMATION
==============================
PMCID: PMC5917375
PMID: 29861691
DOI: 10.1100/tsw.2006.408
Article ID: 149134
Article version: 1
Subjects: Editorial

Editorial: Superficial Bladder Cancer

Kirkali Ziya
Dokuz Eylul University, School of Medicine, Genito-Urinary Group, Inciralti - Izmir, 35340, Turkey
Publication date: 2006
Electronic publication date: 2006 Oct 23
Volume: 6
First page: 2646
Last page: 2646
Copyright: Copyright © 2006 Ziya Kirkali.
Copyright year: 2006
License: This is an open access article distributed under the Creative Commons Attribution License, which permits unrestricted use, distribution, and reproduction in any medium, provided the original work is properly cited.
License URL: https://creativecommons.org/licenses/by/3.0/

==============================
Editorial
Superficial Bladder Cancer
*TheScientificWorldJOURNAL* (2006) 6, 2646
ISSN 1537-744X; DOI 10.1100/tsw.2006.408

TheScientificWorldJOURNAL
www.thescientificworld.com

### Editorial: Superficial Bladder Cancer

Ziya Kirkali
*Dokuz Eylul University, School of Medicine, Genito-Urinary Group, Inciralti - Izmir, Turkey 35340*

**E-mail:** ziya.kirkali@deu.edu.tr

*Published October 23, 2006*

---

Bladder cancer is the fourth common cancer seen in men and accounts for 10% of all cancers seen in developed countries. It is a heterogeneous disease and 70% of the patients are initially seen with the so called "superficial bladder cancer". The true natural history of untreated superficial bladder tumors is not known. While some of these non-invasive tumors do not recur, approximately 80% may have recurrences and up to 20% may progress to muscle-invasive disease and potentially cause metastases and death. World age-standardized mortality rates of bladder cancer vary from 2 to 10 per 100,000. The article by Richard Sylvester delineates the prognostic factors and risk groups for disease recurrence and progression based on the results from the many EORTC GU Group trials conducted in this disease. He states that a new nomenclature that more accurately describes the patient's risk of progression is necessary in order to help determine the best treatment and frequency of follow up.

In his article, Dr. Solsona outlines the importance of new classification of bladder tumors and states that TUR is the treatment for low risk disease followed by intravesical chemotherapy. He cites the randomized trials of the MRC, EORTC and the meta-analysis, and recommends early single intravesical chemotherapy for these patients.

Dr. Van der Meijden addresses the issue of optimal treatment for patients with intermediate and high-risk non-muscle invasive bladder tumors. He explains the pros and cons of both intravesical chemotherapy and immunotherapy with BCG.

TUR remains to be the standard of care for bladder tumors for many years. However, when one looks at the recurrence and progression rates of each subset of non-invasive tumors there are striking differences between different centers and surgeons. This cannot be explained by the different natural behavior of these tumors but implies that the quality of TUR is probably one of the most important issues on recurrence and progression. The technique of TUR and importance of the surgeon is beautifully covered by Drs. Nieder and Manoharan.

Finally Dr.Brausi deals with the new treatment strategies. He covers the issue of drug transport to the bladder, sequential treatment schemes and new drugs.

I would like to thank all authors for their excellent contributions to this Special Issue, which I hope, will please the readers.

---

**This article should be cited as follows:**

Kirkali, Z. (2006) Editorial. Superficial Bladder Cancer *TSW Urology* **1(S2),** 44. DOI 10.1100/tswurol.2006.119.

---

***Corresponding author.**
©2006 with author.
